# Supplementary material for: Comparative genomics provides new insights into the diversity, physiology, and sexuality of the only industrially exploited tremellomycete: Phaffia rhodozyma
Source: BMC Genomics. 2016 Nov 9;17:901. doi: 10.1186/s12864-016-3244-7 (PMC5103461; doi:10.1186/s12864-016-3244-7)
Supplement: Additional file 6: — List of orphan genes with links to PFAM (related to Additional file 1: Table S1). (ZIP 1428 kb) [file 12864_2016_3244_MOESM6_ESM.zip › BLAST_HTML_FTR/G00074_P.html]

BLAST Search Results


```
BLASTP 2.2.27+


Reference:
Stephen F. Altschul, Thomas L. Madden, Alejandro A. Schäffer,
Jinghui Zhang, Zheng Zhang, Webb Miller, and David J. Lipman (1997),
"Gapped BLAST and PSI-BLAST: a new generation of protein database
search programs", Nucleic Acids Res. 25:3389-3402.


Reference for
composition-based statistics:
Alejandro A. Schäffer, L. Aravind, Thomas L. Madden, Sergei
Shavirin, John L. Spouge, Yuri I. Wolf, Eugene V. Koonin, and
Stephen F. Altschul (2001), "Improving the accuracy of PSI-BLAST
protein database searches with composition-based statistics and
other refinements", Nucleic Acids Res. 29:2994-3005.


Database: nr
           71,551,133 sequences; 26,053,659,533 total letters


Query= G00074_P

Length=706
                                                                      Score     E
Sequences producing significant alignments:                          (Bits)  Value

emb|CED84279.1|  Uncharacterized conserved protein [Xanthophyllom...  1266    0.0  


 >emb|CED84279.1| Uncharacterized conserved protein [Xanthophyllomyces dendrorhous]
Length=1109

 Score = 1266 bits (3275),  Expect = 0.0, Method: Compositional matrix adjust.
 Identities = 685/686 (99%), Positives = 686/686 (100%), Gaps = 0/686 (0%)

Query  1    MAKKKTSNNKHKNKMAKVAGATAAVDATATTTDETSAVVEDSVVEESEVIAEAQPETIET  60
            MAKKKTSNNKHKNKMAKVAGATAAVDATATTTDETSAVVEDSVVEESEVIAEAQPETIET
Sbjct  1    MAKKKTSNNKHKNKMAKVAGATAAVDATATTTDETSAVVEDSVVEESEVIAEAQPETIET  60

Query  61   VQVETVQSTEEVVSQPAAVESVPEVIAVEEPLGAPIESTPTAEELESIIDEHKQASSVQV  120
            VQVETVQSTEEVVSQPAAVESVPEVIAVEEPLGAPIESTPTAEELESIIDEHKQASSVQV
Sbjct  61   VQVETVQSTEEVVSQPAAVESVPEVIAVEEPLGAPIESTPTAEELESIIDEHKQASSVQV  120

Query  121  SEVQPEVPTEPVVVQESAVVMEAIPVDASLARELEAIVDDHKVGSASVSVSDVVPETESS  180
            SEVQPEVPTEPVVVQESAVVMEAIPVDASLARELEAIVDDHKVGSASVSVSDVVPETESS
Sbjct  121  SEVQPEVPTEPVVVQESAVVMEAIPVDASLARELEAIVDDHKVGSASVSVSDVVPETESS  180

Query  181  AVTSEEPVEESVAFALNSVVDDHKEKSASVVSVNEVSNQDQEETSIAAVDEEPVEESVAL  240
            AVTSEEPVEESVAFALNSVVDDHKEKSASVVSVNEVSNQDQEETSIAAVDEEPVEESVAL
Sbjct  181  AVTSEEPVEESVAFALNSVVDDHKEKSASVVSVNEVSNQDQEETSIAAVDEEPVEESVAL  240

Query  241  ALNSIVDDHKGKSSSIVSVNEVAVDDREESSAVVNEEPVDASVALALNTVVDDHKGKAPS  300
            ALNSIVDDHKGKSSSIVSVNEVAVDDREESSAVVNEEPVDASVALALNTVVDDHKGKAPS
Sbjct  241  ALNSIVDDHKGKSSSIVSVNEVAVDDREESSAVVNEEPVDASVALALNTVVDDHKGKAPS  300

Query  301  VVSVTEVSNEEPEQDTAAVDPSTALALNTIVDDHKQKNVASTVSVNEVPAEEEQQQRENE  360
            VVSVTEVSNEEPEQDTAAVDPSTALALNTIVDDHKQKNVASTVSVNEVPAEEEQQQRENE
Sbjct  301  VVSVTEVSNEEPEQDTAAVDPSTALALNTIVDDHKQKNVASTVSVNEVPAEEEQQQRENE  360

Query  361  TTEPLDVSVALALNTVVDDHKGKATSTVAVEEVSNEEQEETSTVAPEEPVEESVALALNS  420
            TTEPLDVSVALALNTVVDDHKGKATSTVAVEEVSNEEQEETSTVAPEEPVEESVALALNS
Sbjct  361  TTEPLDVSVALALNTVVDDHKGKATSTVAVEEVSNEEQEETSTVAPEEPVEESVALALNS  420

Query  421  VVDDHKEKSASVVSVNEVVETEETKEVVETPVEPVFAQELEAIVDSHKEASTVSVAEVAA  480
            VVDDHKEKSASVVSVNEVVETEETKEVVETPVEPVFAQELEAIVDSHKEASTVSVAEVAA
Sbjct  421  VVDDHKEKSASVVSVNEVVETEETKEVVETPVEPVFAQELEAIVDSHKEASTVSVAEVAA  480

Query  481  EIVAEVVAEKKQDEVVAPVSAREVVHDEPITTAAVAEPVDAAPAAEDISFEEVKAEDVNE  540
            EIVAEVVAEKKQDEVVAPVSAREVVHDEPITTAAVAEPVDAAPAAEDISFEEVKAEDVNE
Sbjct  481  EIVAEVVAEKKQDEVVAPVSAREVVHDEPITTAAVAEPVDAAPAAEDISFEEVKAEDVNE  540

Query  541  AESDMKKDWVDLALNRELEAIVDSEKENKADVQVQEVAPEEQSAPIPVITNNLASVEAEN  600
            AESDMKKDWVDLALNRELEAIVDSEKENKADVQVQEVAPEEQSAPIPVITNNLASVEAEN
Sbjct  541  AESDMKKDWVDLALNRELEAIVDSEKENKADVQVQEVAPEEQSAPIPVITNNLASVEAEN  600

Query  601  VNRSIEESEVATPVVSETVINQVATEEEAFVAAPTATAADEEEAVKVTSSDTVKNDELVN  660
            VNRSIEESEVATPVVSETVINQVATEEEAFVAAPTATAADEEEAVKVTSSDTVKNDELVN
Sbjct  601  VNRSIEESEVATPVVSETVINQVATEEEAFVAAPTATAADEEEAVKVTSSDTVKNDELVN  660

Query  661  PAEGVMDGKVTESQEEDVVVSKKNKV  686
            PAEGVMDGKVTESQEEDVVVSKKNK+
Sbjct  661  PAEGVMDGKVTESQEEDVVVSKKNKI  686


Lambda      K        H        a         alpha
   0.300    0.117    0.289    0.792     4.96 

Gapped
Lambda      K        H        a         alpha    sigma
   0.267   0.0410    0.140     1.90     42.6     43.6 

Effective search space used: 8082222124412


  Database: nr
    Posted date:  Sep 23, 2015 12:05 AM
  Number of letters in database: 26,053,659,533
  Number of sequences in database:  71,551,133


Matrix: BLOSUM62
Gap Penalties: Existence: 11, Extension: 1
Neighboring words threshold: 11
Window for multiple hits: 40
```
